# The trajectory of anxiety and depressive symptoms and the impact of self-injury: A longitudinal 12-month cohort study of individuals with psychiatric symptoms

**DOI:** 10.1371/journal.pone.0313961

**Published:** 2024-11-21

**Authors:** Olivia Ojala, Maria Å. Garke, Samir El Alaoui, David Forsström, Maria Hedman-Lagerlöf, Simon Jangard, Johan Lundin, Alexander Rozental, Shervin Shahnavaz, Karolina Sörman, Tobias Lundgren, Clara Hellner, Nitya Jayaram-Lindström, Kristoffer N. T. Månsson

**Affiliations:** 1 Department of Clinical Neuroscience, Karolinska Institutet, Centre for Psychiatry Research, Stockholm Health Care Services, Region Stockholm, Stockholm, Sweden; 2 Department of Psychology, Stockholm University, Stockholm, Sweden; 3 Department of Clinical Neuroscience, Division of Psychology, Karolinska Institutet, Stockholm, Sweden; 4 Department of Health, Education and Technology, Luleå University of Technology, Luleå, Sweden; 5 Department of Clinical Psychology and Psychotherapy, Babeș-Bolyai University, Cluj-Napoca, Romania; Taipei Veterans General Hospital, TAIWAN

## Abstract

**Background:**

Individuals reporting self-injury are at greater risk of several adverse outcomes, including suicide. There is reason to be concerned how these individuals cope when stressful life events increase. This study aimed to investigate the trajectories of anxiety and depressive symptoms and the predictive value of self-injury history in individuals with psychiatric symptoms during the unique and stressful conditions of the COVID-19 pandemic.

**Methods:**

In a longitudinal population cohort study (*N* = 1810) ranging from 2020 to 2022, anxiety (measured by Generalized Anxiety Disorder, GAD-7) and depressive symptoms (measured by Patient Health Questionnaire, PHQ-9) were self-reported monthly during 12 months. Latent growth curve models with and without self-reported self-injury history as predictors were conducted.

**Results:**

Overall, anxiety and depressive symptoms decreased from baseline, but remained at moderate severity at follow-up. Individuals reporting suicidal or nonsuicidal self-injury reported significantly higher symptom severity at baseline. In addition, individuals reporting suicidal self-injury demonstrated a slower rate of decline in the symptom load over the course of 12 months.

**Conclusions:**

Over the course of 12 months, anxiety and depressive symptoms decreased in individuals with psychiatric symptoms, but still indicate a psychiatric burden. Individuals with a history of self-injury could be more vulnerable in face of stressful conditions such as those experienced during the COVID-19 pandemic.

## Introduction

There is reason to be concerned about how individuals with psychiatric disorders, especially those with a history of self-injury, cope in a societal crisis. Self-injury has a lifetime prevalence ranging from 24% [1] to 55% [2] in individuals with psychiatric symptoms, and can be triggered by stressful life events and hopelessness [3,4]. During societal crisis, such as infectious disease epidemics, the prevalence of self-injury seems to be similar to non-epidemic periods [5]. Self-injury is one of the criteria for borderline personality disorder (BPD) but many of those engaging in self-injury do not fulfil criteria for BPD [6–8]. Rather, self-injury can be present in comorbidity with a wide range of psychiatric disorders, including internalizing, externalizing, substance use, and neurodevelopmental disorders [6–8]. Furthermore, self-injury with and without the intention to die is a risk factor for several adverse outcomes, such as restrictive inpatient care, high rates of anxiety and depression, and suicide [3,9,10]. Conversely, adverse mental health also increases the risk of self-injury [3,4]. Individuals with a history of self-injury often display difficulties regulating emotions adaptively [11,12] which is also associated with anxiety and depressive symptoms [13]. Thus, in times of crisis when difficult emotions arise, those with a history of self-injury may have greater difficulties handling such emotions and, in turn, experience increased anxiety and depressive symptoms. This can create a vicious cycle as self-injury ca serve as a maladaptive coping strategy for unwanted emotional states, such as depression and anxiety, which then triggers further self-injury [12].

The COVID-19 pandemic significantly impacted the health care system, which in turn caused significant concerns about how this impacted individuals experiencing psychiatric symptoms [14]. During the early phase of the pandemic, there were alarming reports on anxiety and depressive symptoms increasing in the general population [15,16] and in individuals with a history self-injury [15,17] but later decreased in the general population [18]. Similar decreases has been detected in individuals with psychiatric symptoms in several longitudinal studies [18–20] although one study showed unaltered levels of anxiety and depressive symptoms [21]. However, knowledge about how psychiatric symptoms developed over more than ten months, beyond the first year of the COVID-19 pandemic [18–21], and in individuals with a history of self-injury, is limited.

Consequently, whether the intention of self-injury has any bearing on the trajectories of anxiety and depressive symptoms is unknown. Nonsuicidal and suicidal self-injury could be separated by intention, but also by rate and lethality [22]. Additionally, individuals with psychiatric disorders with a history of suicidal behaviour seem to experience a lower capacity for successful adaption to changes [23]. This lower degree of resilience is troublesome in a health crisis, such as the COVID-19 pandemic, which comprises a state of constant societal changes. More research is needed to increase our understanding of potential additional care needs, for individuals with a history of nonsuicidal and suicidal self-injury during a societal crisis.

In summary, it remains unclear how anxiety as well as depressive symptoms developed over more extended periods in a psychiatric population as the pandemic proceeded and how self-injury history impacted the trajectories. A long-term investigation, including several waves of COVID-19, could capture the impact of a persistent pandemic and its subsequent restrictions. It could also provide information about potential treatment needs during longer periods of societal crisis. The current longitudinal study thus intended to monitor individuals with psychiatric symptoms monthly over 12 months, ranging from 2020 to 2022.

Our study has two aims: The first was to explore the overall trajectory of anxiety and depressive symptoms. Although anxiety and depressive symptoms tend to overlap, they differ in some aspects [24]. For instance, anxiety tends to be more oriented towards the future while depression often relates more to the past; depression typically involves a relative absence of positive affect, and anxiety is characterized by hyperarousal. [24] Monitoring both anxiety and depressive symptoms was stated as research priorities of the COVID-19 pandemic [14]. The second aim was to investigate if nonsuicidal and suicidal self-injury were associated with higher baseline symptom severity and explore potential differences in the trajectories based on self-injury history. We expected a history of both nonsuicidal and suicidal self-injury to be predictive of elevated symptom severity in this specific population.

## Materials and methods

### Study design

The study was part of a longitudinal online survey study [25] monitoring individuals with self–reported psychiatric symptoms or disorders over a total of 12 months during the COVID-19 pandemic. The recruitment period and gathering of baseline data was done between the 2^nd^ of July 2020 and the 21^st^ of June 2021. Follow-up data was collected from August 2020 to May 2022. The project was approved by the Swedish Ethical Review Authority (registration number 2020–02798) and all participants gave written informed consent electronically.

### Participants

To recruit as many participants in the target group as possible across a short time frame covering the pandemic development, only a few eligibility criteria applied. Inclusion criteria were age 18 or older, living in Sweden, and having current or lifetime experience of psychiatric symptoms or disorders. There were no eligibility criteria of current or previous healthcare contacts or treatment for psychiatric symptoms. In total, 5936 individuals consented to longitudinal follow-up, and 4118 (69%) provided demographic and clinical data at baseline relevant to this study. Participants with complete outcome data at baseline, as well as outcome measure data for at least three follow-ups across the rest of the study period (i.e., having participated in one follow-up from months 1–4, one from months 5–8, and one from months 9–12, respectively), were included and analysed in the current study. The resulting sample consisted of 1810 adults aged 18–81 years who contributed with data at least once every quarter of the 12-month follow-up. The participant selection was based on the fact that at least four measurements are recommended for an efficient latent growth curve model [26]. Selecting participants based on these criteria, yielded one of the largest sample sizes. It was preferred based on the intention of the current study to explore outcomes over a longer time frame.

### Procedures

Study recruitment started in July 2020 and ended in June 2021. Participants were recruited through advertisements placed at psychiatry and primary care clinics and posted on social media and via patient organizations. Advertisements stated that the study aimed to include individuals who had experienced mental illness either currently or previously. The advertisements were adapted to increase the sample’s representativeness concerning age, gender, and country of origin (e.g., advertising in different languages and with different images). Public awareness of the project was facilitated through presentations in the Swedish news (on both radio and television) and presentations in patient and caregiver associations. Data was collected using online surveys in REDCap [27,28] (accessed via a webpage). Participants responded to several online questionnaires about mental health (as described in detail elsewhere) [25], including those used in the current study. After they consented to participate and responded to the baseline survey, they were invited via email to a follow-up survey every 30 days for 12 months (average number of follow-ups is presented in the *Results* section). The emails contained a personal link to the given follow-up survey; once a survey was completed, it could not be completed again. For the follow-up invitations, participants who did not fill out the monthly survey received daily reminders via email, for up to five days. A proportion of participants were randomly assigned a gift card worth 150 SEK (approximately €15) for their participation.

### Measures

#### Demographic information

At baseline, participants self-reported age, gender, occupation, country of origin, and educational level. Furthermore, the participants were asked to report all lifetime psychiatric disorders that had been diagnosed with from a list of common psychiatric and neurodevelopmental disorders.

#### Outcomes

Depressive symptoms in the past two weeks were measured with the Patient Health Questionnaire (PHQ-9) [29], a psychometrically sound [30] and widely used 9-item questionnaire containing items rated on a 4-point scale ranging from 0 (“Not at all”) to 3 (“Nearly every day”). A summary score of 0–4 indicates “Minimal or no depression, 5–9 “Mild depression”, 10–14 “Moderate depression”, 15–19 “Moderately severe depression”, and 20–27 “Severe depression”. In the current study, internal consistency was Cronbach’s α = .88 at baseline. Item 9 (“Thoughts that you would be better off dead or of hurting yourself in some way”) was used in sensitivity analysis as a proxy measure for self-injury thoughts (similar to another study) [31]. The item was dichotomized with 0 representing a response of “Not at all” and 1 representing all other response options (i.e., “Several days”, “More than half the days”, “Nearly every day”).

Anxiety symptoms during the past two weeks were measured with the Generalized Anxiety Disorder 7-item scale (GAD-7) [32], a psychometrically sound [32] and widely used questionnaire containing items rated on a 4-point scale ranging from 0 (“Not at all”) to 3 (“Nearly every day”). A summary score of 0–4 indicates “None”, 5–9 “Mild anxiety”, 10–14 “Moderate anxiety”, and 15–21 “Severe anxiety”. In the current study, internal consistency was Cronbach’s α = .91 at baseline.

#### Self-injury predictors

Four questions about self-injury were adapted from a questionnaire developed for the UK Biobank [33], separating nonsuicidal and suicidal self-injury (as they can be seen as separate entities [22,34]). Participants were asked if they ever had deliberately harmed themselves (e.g., through cutting, biting, or hitting themselves) without the intention to end one’s life. A “yes” was coded as presence, and a “no” was coded as absence of nonsuicidal self-injury. In addition, participants were asked if they had ever deliberately harmed themselves (e.g., through cutting, biting, or hitting themselves or taking pills) with the intention to end one’s life. A “yes” was coded as presence, and a “no” was coded as absence of suicidal self-injury. If participants responded “yes” to a question about lifetime engagement, they were instructed to a indicate recent engagement (i.e., past four weeks; yes or no). The self-injury questions were followed by instructions on what to do in case of self-injury impulses and contact information for healthcare and relevant services.

### Data preparation and statistical analyses

Data preparation and statistical analyses were carried out in R version 4.1.3 [35]; see S1 Appendix for package specifications. In terms of data quality, invalid answers were checked for and one answer, with a participant reporting age >200, was assessed to be invalid. All participants reported a valid email address. All statistical analyses reported included full *p*-values and 95% confidence intervals. Descriptive statistics were reported, including means, standard deviations, and proportions. For the growth curves, all available data was used per our selection criteria (at least four repeated data points in the outcome variable across time as described in the *Participants* section), which the statistical model allows for [36]. Missing data was handled through maximum likelihood estimation in the growth curves [26]. Anxiety and depressive symptoms scores were, on inspection, approximately normally distributed across all measurement points and had skewness and kurtosis values below recommended thresholds [37]. Pseudo-R^2^ was used to estimate effect sizes, approximating the proportion of residual variance explained by the predictor in the model [38]. It was derived by subtracting the residual variance from the model with the predictor from the residual variance from the model without the predictor, divided by the residual variance from the model without the predictor.

Two growth curve models were specified to investigate change over time in anxiety and depressive symptoms, respectively (see S1 Appendix for full model specification). Upon visual inspection of the average change over time at the levels of groups and among individual cases, the trend was distinctly linear. Consequently, linear models were fitted in a stepwise process, first adding time as a predictor of the outcome (Aim 1; unadjusted models). In the next set of models (Aim 1; adjusted models), covariates were added, including time in days since individual study start (to control for the different dates that participants entered the study as this varied over 11 months), age, gender, and educational level. The rationale for adding these covariates was that age, gender, and educational level have been shown to have an impact on the initial level of anxiety and depressive symptoms during COVID-19, as well as the change over time [18]. As a sensitivity analysis, changes in self-injury thoughts over time were investigated with an unadjusted logistic growth curve including only time as a predictor. No covariates or self-injury predictors were included in this sensitivity analysis due to model non-convergence (likely a combination of lack of variability in the binary outcome and excessive model complexity). The rationale for this sensitivity analysis was to check if this specific item (i.e., self-injury thoughts) followed the same pattern as the full depression scale.

For aim 2, the growth curves were extended to include the predictors of nonsuicidal self-injury and suicidal self-injury. These models tested the predictive value of self-injury (binary measure: yes/no) on the change in anxiety and depressive symptoms over time (see S1 Appendix for full model specification). The self-injury predictors were added in separate models (due to the overlap among those engaging in nonsuicidal and suicidal self-injury). The reference group in the model with nonsuicidal self-injury were individuals without nonsuicidal self-injury, and for suicidal self-injury, individuals without suicidal self-injury. All models included the self-injury predictor and its interaction with time as fixed effects both with and without adjustment for the covariates mentioned above. Cluster-robust confidence intervals were computed which resulted in near-identical estimations. Thus, the original models were presented. All growth curves included random slopes and intercepts and were fitted using restricted maximum likelihood. To further test the robustness of the findings, post-hoc sensitivity analyses were performed for models with significant time-predictor interactions. We aimed to assess the potential influence of clinical levels of anxiety and depressive symptoms (measured with GAD-7 and PHQ-9) at baseline on the predictive effect of self-injury. The rationale for these analyses was to explore whether the results were specific to self-injury or explained by greater clinical severity. Two additional binary covariates were added to the adjusted model, representing the absence or presence of clinical anxiety and depression (total scores ≥ 15 for GAD-7 [cut-off for severe anxiety [32] in the absence of proxy for diagnosed anxiety] and ≥ 14 for PHQ-9 [a proxy for the presence of major depression [39]]).

## Results

Demographic and clinical characteristics are reported in Table 1 for the whole study sample. At baseline, 711 (39%) participants reported no history of self-injuries. In contrast, 571 (32%) participants reported a history of nonsuicidal self-injury only, 442 (24%) participants reported a history of both nonsuicidal and suicidal self-injury, and 86 (5%) participants reported a history of suicidal self-injury only. For individuals reporting a history of suicidal self-injury, 44 out of 528 (8.3%) reported suicidal self-injury the past four weeks at baseline. For those reporting a history of nonsuicidal self-injury, 254 out of 1013 (25.1%) reported nonsuicidal self-injury the past four weeks at baseline. Demographics and clinical characteristics of the different self-injury groups (i.e., suicidal self-injury, nonsuicidal self-injury, or no self-injury; participants with a history of both suicidal and nonsuicidal self-injury are included in both self-injury groups, aligning with the main analyses) are presented in S1 Table.

**Table 1:** Demographic and clinical characteristics of study sample (*N* = 1810).

Variable	*M (SD)*	Range
*Age*	38.07 (12.69)	18–81
*Self-injury*	***n***	**%**
Lifetime suicidal self-injury	528	29
Lifetime nonsuicidal self-injury	1013	56
Recent suicidal self-injury (past 4 weeks)	44	2
Recent nonsuicidal self-injury (past 4 weeks)	254	14
*Gender*		
Male	353	20
Female	1357	75
Other	100	5
*Birthplace*, *Sweden*	1691	93
*Educational level (highest)*		
Elementary school	98	5
High school	577	32
University	1135	63
*Employment status before COVID-19* ^a^		
Student	398	22
Unemployed	179	10
Part-time employee/hourly employee	433	24
Full-time employee	722	40
Retired	175	10
*Lifetime psychiatric disorder* ^,*a*,*b*,*c*^		
Bipolar and related disorders	351	19
Major depressive disorder	1480	82
Anxiety disorders		
Social	336	18
Panic	677	37
Generalized	770	43
Trauma- and stressor-related disorders	377	21
Feeding and eating disorders	407	22
Neurodevelopmental disorders	381	21

*Note*. All variables are self-rated by participants at baseline. The “Other” category in gender included response options non-binary, prefer to self-define, and prefer not to answer; ^a^Multiple response options allowed; ^b^The reported disorders are compiled in accordance with the categorization in the Diagnostic and Statistical Manual of Mental Disorders (DSM-5 [40]).

^c^In total, 35 (1.9%) individuals responded “Do not know” and 106 (5.8%) responded that none of the diagnostic categories (corresponding to psychiatric disorders and neurodevelopmental disorders in the DSM-5) matched them.

The average number of repeated measures, including the baseline measurement, for each participant was 11 (median = 12, min, max = 4, 13; minimum number of repeated measures was four in the current study), resulting in approximately 20 000 observations available for analysis (see Table 2 for details including number of observations and descriptive statistics for the outcomes on a month-by-month basis). Baseline demographics and clinical characteristics for individuals partaking in the present longitudinal study were comparable to those not adhering to multiple follow-ups across the study period. However, statistically significant differences were identified in several aspects (S2 Table).

**Table 2:** Descriptive statistics of anxiety and depressive symptoms and self-injury thoughts across all time points (all available data in study sample).

	PHQ-9		GAD-7		Self-injury thoughts^a^	
**Time point**	*n*	*M* (SD)	*n*	*M* (SD)	*n*	*n* (%)
Baseline	1810	14.57 (6.61)	1810	11.50 (5.72)	1810	940 (52)
1 month	1645	13.37 (6.52)	1640	10.17 (5.64)	1645	769 (47)
2 months	1600	13.22 (6.67)	1599	10.15 (5.78)	1600	724 (45)
3 months	1605	13.07 (6.80)	1603	10.12 (5.92)	1605	751 (47)
4 months	1592	13.05 (6.88)	1592	10.09 (5.92)	1592	730 (46)
5 months	1617	12.87 (6.94)	1617	10.01 (5.97)	1617	755 (47)
6 months	1531	12.94 (7.04)	1521	10.16 (5.96)	1531	696 (45)
7 months	1501	12.58 (6.98)	1495	9.67 (5.85)	1501	653 (44)
8 months	1512	12.28 (6.98)	1512	9.52 (6.00)	1512	654 (43)
9 months	1516	12.19 (6.98)	1514	9.55 (6.05)	1516	688 (45)
10 months	1429	12.03 (7.00)	1425	9.42 (6.05)	1429	613 (43)
11 months	1353	12.01 (6.95)	1352	9.40 (6.02)	1353	568 (42)
12 months	1275	12.45 (6.99)	1268	9.78 (6.19)	1275	565 (44)

*Note*. PHQ-9 = Patient Health Questionnaire, GAD-7 = Generalized Anxiety Disorder 7-item scale.

^a^Self-injury thoughts = based on PHQ-9 item 9.

Baseline PHQ-9 and GAD-7 scores indicated moderate and moderately severe depression and anxiety, respectively (adjusting for time since study start, age, gender, and education level; S1 Fig). Scores decreased on average with 0.17 (PHQ-9) and 0.12 (GAD-7) points each month and resulted in a total predicted average decrease of 2.0 (PHQ-9) and 1.5 (GAD-7) points (see also S3 and S4 Tables and S1 Fig) indicating moderate levels at the 12-month follow-up. Time as a predictor explained 16% (pseudo R^2^) of the variance in both anxiety and depressive symptoms. Hence, time explained a smaller proportion of the variance in symptom reduction. In addition to anxiety and depressive symptoms, self-injury thoughts also decreased slightly over time, with a lower odds ratio (i.e., <1) of reporting such thoughts month-by-month (S5 Table). Proportions of individuals reporting self-injury thoughts month-by-month, separated based on self-injury history, are found in S6 Table.

**Fig 1:**
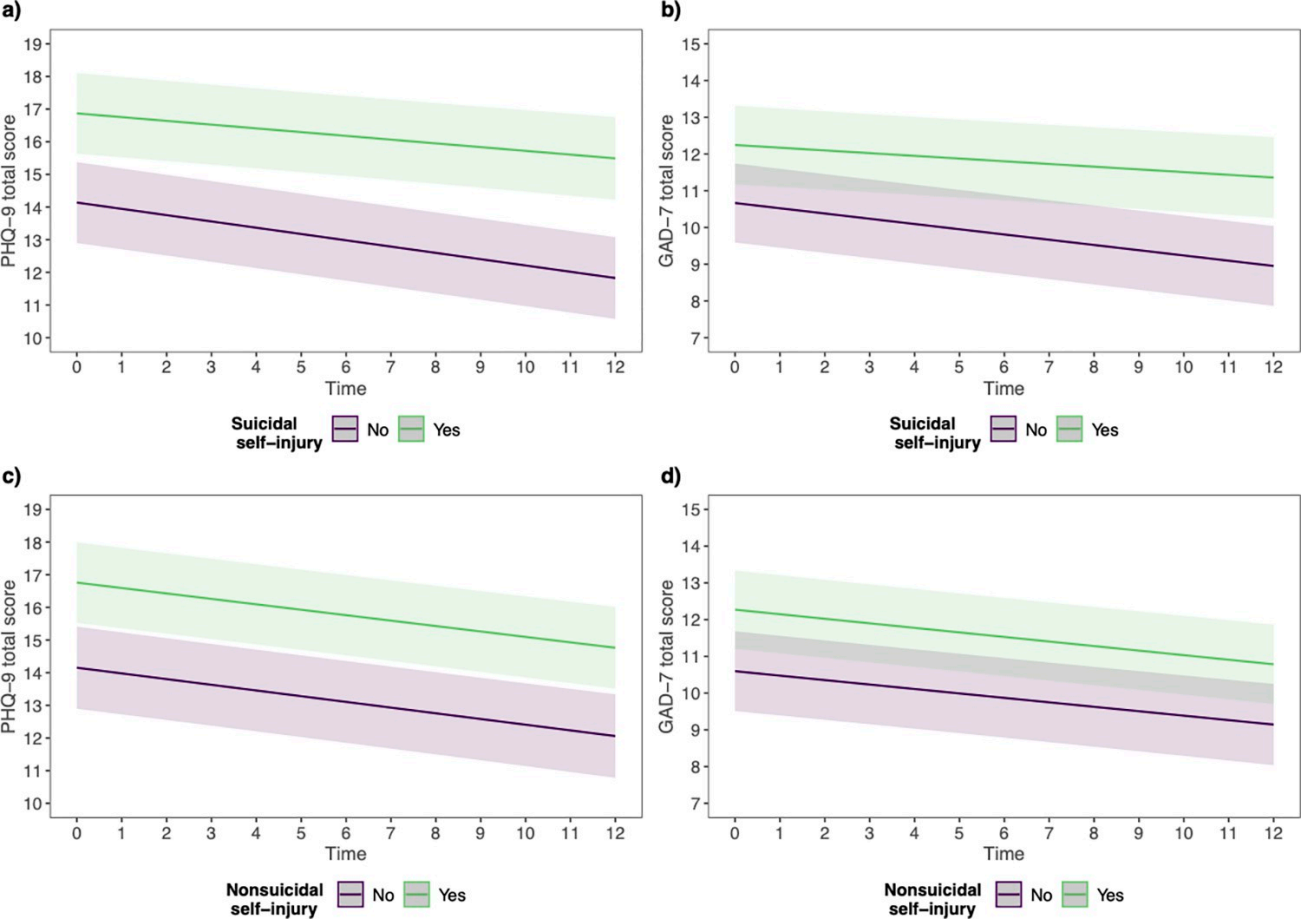
Predicted trajectories of anxiety and depressive symptoms of suicidal and nonsuicidal self-injury. *Note*. All figures represent predicted trajectories of anxiety and depression with 95% confidence intervals starting at baseline (0) and the 12 months follow-up. a) Predicted PHQ-9 scores among individuals with and without a history of suicidal self-injury, b) Predicted GAD-7 scores among individuals with and without a history of suicidal self-injury, c) Predicted PHQ-9 scores among individuals with and without a history of nonsuicidal self-injury, d) Predicted GAD-7 scores among individuals with and without a history of nonsuicidal self-injury.

As expected, a history of suicidal self-injury was a significant predictor of higher baseline anxiety and depressive symptoms scores (see Tables 3 and S7). Further, there were significant interactions with time in both trajectories (Table 3). These interactions remained significant after controlling for baseline symptom severity, time since study start, age, gender, and education level. As reported in Fig 1A and 1B and Table 3, anxiety and depressive symptoms in individuals reporting suicidal self-injury decreased at a slower rate than those reporting no suicidal self-injury (Table 3, row”Suicidal self-injury x Time interaction”). The significant interaction effect between time and suicidal self-injury pertained while also controlling for clinical levels of anxiety and depressive symptoms in the post hoc sensitivity analysis (see S8 Table). Furthermore, predicted average scores at 12-month follow-up for individuals reporting suicidal self-injury indicated moderate anxiety and moderately severe depression, in contrast to individuals reporting no suicidal self-injury where mild anxiety and moderate depression were indicated (see Fig 1A and 1B).

**Table 3:** Results from adjusted growth curve models with all self-injury predictors of anxiety and depressive symptoms trajectories.

	Depressive symptoms				Anxiety symptoms			
	*b*	95% CI	*SE*	*p*	*b*	95% CI	*SE*	*p*
**Suicidal self-injury**								
Suicidal self-injury	2.73	2.14, 3.33	0.30	< .001	1.58	1.06, 2.10	0.26	< .001
x Time interaction	0.08	0.03, 0.13	0.02	.001	0.07	0.03, 0.11	0.02	.001
**Nonsuicidal self-injury**								
Nonsuicidal self-injury	2.61	2.01, 3.20	0.30	< .001	1.67	1.16, 2.19	0.26	< .001
x Time interaction	0.01	-0.04, 0.05	0.02	.714	-0.00	-0.04, 0.04	0.02	.889

*Note*. Adjusted for age, gender, educational level, and time since study start.

Individuals with a history of nonsuicidal self-injury also reported significantly higher baseline anxiety or depressive symptoms (see Tables 3 and S9 and Fig 1C and 1D). The interaction between nonsuicidal self-injury and time was non-significant in both trajectories, meaning the trajectories of PHQ-9 and GAD-7 did not differ significantly based on the history of nonsuicidal self-injury. Furthermore, predicted average scores at 12-month follow-up for individuals reporting nonsuicidal self-injury indicated moderate anxiety and moderately severe depression, in contrast to individuals without nonsuicidal self-injury where mild anxiety and moderate depression were indicated (see Fig 1C and 1D).

Age, gender, and education level showed significant associations with both baseline PHQ-9 and GAD-7 in all the adjusted primary models (i.e., both in the models with and without nonsuicidal and suicidal self-injury predictors). However, the effect of age on depressive symptoms in the model with nonsuicidal self-injury was non-significant (S9 Table). Thus, in nearly all cases, younger individuals, with lower education level, and females (relative to males) reported higher baseline PHQ-9 and GAD-7 scores, respectively (S3 and S4 Tables, S7–S9 Tables).

## Discussion

In the current longitudinal study, 1810 individuals with psychiatric symptoms were monitored monthly, for one year from 2020 to 2022. One key finding was that both anxiety and depressive symptoms decreased over time but at a significantly slower rate for individuals reporting a history of suicidal self-injury. Less decrease in symptom load was evident regardless of baseline levels of anxiety and depressive symptoms. Another relevant clinical finding was that individuals reporting nonsuicidal or suicidal self-injury had significantly higher baseline values of anxiety and depressive symptoms as compared to those negating such behaviours.

The findings from the present longitudinal study add to previous literature demonstrating a slight decrease in anxiety and depressive symptoms in individuals with psychiatric symptoms during a pandemic [18–20]. The collective literature reports an observed decrease in several countries in Europe. However, the three previously conducted longitudinal studies only monitored individuals up to four time points [19,20] and, at most, during five months [18–20]. One previous longitudinal study monitored individuals with psychiatric symptoms up to ten months during the first year of the pandemic and showed no significant change over time [21]. With the current longitudinal study, we extend previous research by showing a decrease with monthly measurements over 12 months, beyond the first year of the pandemic in individuals with various psychiatric profiles. In addition to anxiety and depressive symptoms, self-injury thoughts (i.e., PHQ-9 item 9) also decreased over the course of 12 months. Hence, the prevalence of self-injury thoughts could be expected to follow the same trajectory as anxiety and depressive symptoms in individuals with psychiatric symptoms during a societal crisis. Decreases in mental health symptoms over time are not unique to the COVID-19 pandemic but have also been observed in other societal crises, such as the September 11th attacks [41]. Although this study cannot answer why a decrease in symptoms is found in all models, hypothetical reasons could include the effects of monitoring mood and symptoms regularly [42] or regression towards the mean. However, it is unlikely that regression towards the mean is the only reason; those reporting suicidal self-injury reported more severe psychiatric symptoms at baseline but smaller decreases. If regression towards the mean was to explain the results, larger decreases would be expected rather than smaller decreases. Additional hypothetical reasons could be that some individuals found it easier to manage the challenges the pandemic entailed, the longer it persisted as some routines became more established in Sweden (i.e., work and social life restrictions), or that some individuals received the needed treatment for psychiatric symptoms offered by health care. The clinical relevance of the significant decrease in anxiety and depressive symptoms should, however, be addressed. The decreases only correspond to one or a few points change, on the PHQ-9 or GAD-7 self-reported questionnaires. Decreases of small magnitude (i.e., only a few points change on the PHQ-9 and GAD-7) echo findings from a previous longitudinal study conducted during the COVID-19 pandemic [18]. In this study, moderate levels of anxiety and depressive symptoms were reported during the five months that individuals with psychiatric symptoms were monitored [18]. Our results point to similar severity levels but over a longer period. It is interesting to note is that similar severity levels thus were observed in Sweden and United Kingdom; unlike United Kingdom and other European countries, Sweden did not have lockdown periods but had recommendations for physical distancing, avoiding public places, and wearing masks. There were also occasional restrictions on restaurant and café opening hours, as well as visitor limits at public places. In summary, individuals with psychiatric symptoms could experience moderate levels of anxiety and depressive symptoms over an extended period during a societal crisis, even without lockdown periods.

The present findings indicate that a history of suicidal self-injury negatively impacts the trajectory of anxiety and depressive symptoms during a stressful life event such as pandemic. The results from the post-hoc sensitivity analyses suggest that the findings do not seem to be explained by high clinical severity at baseline (as measured by clinical levels of anxiety indicated by GAD-7 and depression indicated by PHQ-9 at baseline). A previous study performed during the COVID-19 pandemic [20] reported that the presence of a depressive disorder did not impact the trajectory of anxiety and depressive symptoms. In contrast, the presence of an anxiety disorder predicted a greater decrease [20]. Thus, the adverse impact self-injury history has on the trajectories could differ from the impact of other clinical predictors (i.e., high levels of anxiety or depressive symptoms). Suicidal self-injury entails a severe mental health state that can have several adverse consequences years after the self-injurious behaviour occurs [9,10]. One can speculate that responses in a crisis from individuals with such history could, on a group level, differ compared to those without such history. One could also consider the role of vulnerability; individuals with a history of suicidal behaviour report a lower capacity for successful adaption to acute and chronic changes [23]. As the COVID-19 pandemic forced many societal changes (e.g., cancelled events and restricted physical contact), it could be that this specific subgroup of individuals experienced more difficulties adapting to such changes, thereby negatively impacting their well-being. To what extent these results are unique to the COVID-19 pandemic context or could generalize to other contexts remains unknown. However, given that there is an increased risk of diagnosed anxiety and depression for adolescents in the general population years after engagement in self-injury [9], it may be that our results could generalize to the non-pandemic context and indicate a worse trajectory on the symptom level. As expected, a history of nonsuicidal or suicidal self-injury was associated with higher baseline levels of anxiety and depressive symptoms. Higher levels of anxiety and depression in individuals reporting self-injury have also been demonstrated in previous studies conducted during the pandemic with community samples [15,17]. In addition to previous studies, the current study shows these associations in individuals with a history of psychiatric symptoms and, importantly, for both nonsuicidal as well as suicidal self-injury. The results imply that different types of self-injury histories can be seen as severity markers beyond other psychiatric symptoms in times of a societal crisis. Future research could extend the knowledge by examining the different self-injury groups separately (i.e., suicidal or nonsuicidal self-injury only or both nonsuicidal and suicidal self-injury). Furthermore, examining if the predictive value of self-injury affects trajectories at different ages and genders (i.e., using a three-way interaction) can provide valuable insight into whether certain groups are at risk of experiencing more adverse trajectories during times of societal crisis.

There are some important limitations to highlight. Only participants that contributed with at least baseline and three follow-ups across 12 months were included. This subgroup constitutes 44% of all participants who responded to the demographic and clinical questions at baseline and could potentially be affected by selection bias. The included relative to the excluded sample showed a similar proportion of suicidal self-injury history (29% versus 30%), and diagnostic profiles (S2 Table). The average mean score of baseline anxiety and depressive symptoms (PHQ-9: 14.6 versus 15.8; GAD-7: 11.5 versus 12.2) were slightly different but only differed with approximately one point. Furthermore, in the included relative to the excluded sample, slightly fewer had engaged in nonsuicidal self-injury (56% vs 59%), were males (20% versus 25%), and fewer had university education (63% versus 48%; S2 Table). Convenience sampling was used, and although efforts were made to increase the sample’s representativeness, we were not able to recruit enough males and individuals originating from countries other than Sweden. Furthermore, the number of data observations decreased with time in the current study sample, introducing another potential risk of selection bias. However, it should be noted that 70% of the included participants provided data at the 12-month follow-up, and we used a model in which all data points were used. The presence and character of psychiatric symptoms or disorder(s) were self-reported and not formally evaluated by a clinician. The generalizability to patients in a treatment setting is thus unknown. On the other hand, the findings could be applicable to a broader group of individuals experiencing psychiatric symptoms. Furthermore, reliable and valid measures of self-reported anxiety and depressive symptoms (i.e., PHQ-9 and GAD-7) demonstrated high levels of such symptoms as well as a prevalence of self-injury behaviours. These results indicate a psychiatrically burdened sample. Although the outcome measures have shown good psychometric properties, self-report bias could still have a negative impact on the validity of the findings. Lastly, we do not have pre-pandemic information regarding this sample. Hence, we cannot know if symptom severity levels or trajectories might have changed from before to during the pandemic. Previous literature suggests that there were similar or elevated levels of depressive and anxiety symptoms during the pandemic [15,16].

Even though this study did not focus on intervention, the findings could still have some clinical implications of interest. The lasting psychiatric burden in this vulnerable population indicate the need for early, accessible, and acceptable interventions that could be put in place during times of crisis. In addition, individuals with self-injury may need additional resources, given the elevated levels of symptomatology compared to individuals without self-injury. A history of suicidal self-injury history could indicate a need for healthcare services to identify and carefully monitor these high-risk individuals as early as possible. Potentially, these individuals might need additional support to successfully adapt to sudden changes entailed by personal or societal crises.

## Conclusions

Over the course of 12 months, anxiety and depressive symptoms decreased in individuals with psychiatric symptoms, but still indicate a psychiatric burden. Individuals with a history of self-injury could be more vulnerable in face of stressful conditions such as those experienced during the COVID-19 pandemic.

## Supporting information

S1 Appendix Methods supplement. (PDF)

S1 Table Demographic and clinical characteristics of study sample at baseline by endorsement of self-injury. (PDF)

S2 Table Demographic and clinical characteristics of study sample (N = 1810) vs. participants consenting to follow-up providing demographic and outcome data at baseline (N = 2308). (PDF)

S3 Table Results from growth curve models with depressive symptoms (PHQ-9) as outcome. (PDF)

S4 Table Results from growth curve models with anxiety symptoms (GAD-7) as outcome. (PDF)

S5 Table Results from logistic growth curve models with self-injury thoughts as outcome. (PDF)

S6 Table Proportion of individuals reporting self-injury thoughts across all time points by endorsement of self-injury (all available data in study sample). (PDF)

S7 Table Results from unadjusted and adjusted growth curve models with suicidal self-injury as a predictor of depression and anxiety trajectories. (PDF)

S8 Table Results from post hoc sensitivity analyses including initial level of anxiety and depressive symptoms in the adjusted growth curve models with history of suicidal self-injury as a predictor of depression and anxiety trajectories. (PDF)

S9 Table Results from unadjusted and adjusted growth curve models with nonsuicidal self-injury as a predictor of depression and anxiety trajectories. (PDF)

S1 Fig Predicted trajectories of depressive and anxiety symptoms, adjusted and unadjusted for covariates. (PDF)

## Abbreviations

GAD: Generalized Anxiety Disorder
PHQ: Patient Health Questionnaire
