# Supplementary material for: The trajectory of anxiety and depressive symptoms and the impact of self-injury: A longitudinal 12-month cohort study of individuals with psychiatric symptoms
Source: PLoS One. 2024 Nov 21;19(11):e0313961. doi: 10.1371/journal.pone.0313961 (PMC11581223; doi:10.1371/journal.pone.0313961)
Supplement: S1 Appendix — (PDF) [file pone.0313961.s001.pdf]

S1 Appendix. Methods supplement

R-package specification
For preparing and analysing data, the following R-packages were used: tidyverse for data cleaning and visualization[1], psych for descriptive statistics[2], lme4 for growth curve modeling[3], clubSandwich for cluster-robust confidence interval estimation[4], as well as sjPlot and ggpubr for visualization.[5,6]

Growth curve model specifications
Equation for growth curve model investigating change over time in anxiety and depressive symptoms (excluding covariates):

$$Y_{ij} = \beta_0 + \beta_{1j}t_{ij} + u_{0j} + u_{1j}t_{ij} + \varepsilon_{ij}$$

In above equation, Y_{ij} is the outcome at occasion *i* for individual j, β_0 is the overall intercept of the dependent variable (GAD-7 or PHQ-9 score), β_{1j} is the slope (in this context the growth rate) for individual j, t_{ij} is the time point (occasion), u_{0j} and u_{1j} refers to the individual random effects (intercept and slope) for time, while e_{ij} refers to the residual variance.[7]

Equations for growth curve model investigating effect of self-injury and interactions with time (excluding covariates):

$$Y_{ij} = \beta_0 + \beta_{1j}t_{ij} + \beta_2 SI_j + \beta_3 t_{ij} SI_j + u_{0j} + u_{1j}t_{ij} + \varepsilon_{ij}$$

In above equation, Y_{ij} is the outcome at occasion *i* for individual j, β_0 is the overall intercept of the dependent variable (GAD-7 or PHQ-9 score), β_{1j} is the slope (in this context the growth rate) for individual j, t_{ij} is the time point (occasion), $\beta_2 SI_j$ is the effect of self-injury (SI), $\beta_3 t_{ij} SI_j$ is the effect of the interaction between self-injury (SI) and time, u_{0j} and u_{1j} refers to the individual random effects (intercept and slope) for time, while e_{ij} refers to the residual variance.[7]

References

1. Wickham H, Averick M, Bryan J, et al. Welcome to the tidyverse. *Journal of Open Source Software* 2019; **4**(43): 1686.
2. Revelle W. psych: Procedures for psychological, psychometric, and personality research. Evanston, Illinois; 2019.
3. Bates D, Mächler M, Bolker B, Walker S. Fitting Linear Mixed-Effects Models Using lme4. *Journal of Statistical Software* 2015; **67**(1): 1 - 48.
4. Pustejovsky J. clubSandwich: Cluster-robust (sandwich) variance estimators with small-sample corrections. 2022.
5. Lüdecke D. sjPlot: Data visualization for statistics in social science. 2021.
6. Kassambara A. ggpubr: 'ggplot2' based publication ready plots. 2020.
7. Steele F. Multilevel modelling of repeated measures data. *LEMMA VLE Module* 2014; **15**: 1–64.
