# Supplementary material for: The trajectory of anxiety and depressive symptoms and the impact of self-injury: A longitudinal 12-month cohort study of individuals with psychiatric symptoms
Source: PLoS One. 2024 Nov 21;19(11):e0313961. doi: 10.1371/journal.pone.0313961 (PMC11581223; doi:10.1371/journal.pone.0313961)
Supplement: S1 Table — (PDF) [file pone.0313961.s002.pdf]

S1 Table

Demographic and clinical characteristics of study sample at baseline by endorsement of self-injury

	**Suicidal self-injury**[a] (*n* = 528)	**Nonsuicidal self-injury**[a] (*n* = 1013)	**No self-injury** (*n* = 711)
**Variable**	*M (SD)*	*M (SD)*	*M (SD)*
Age M *(SD)*	36.58 (12.40)	33.48 (10.36)	43.38 (13.07)
PHQ-9 total score	16.71 (6.58)	15.82 (6.41)	12.73 (6.39)
GAD-7 total score	12.58 (5.67)	12.42 (5.48)	10.30 (5.77)
	n (%)	*n (%)*	*n (%)*
Self-injury thoughts	366 (69)	643 (63)	251 (35)
Self-injury			
Lifetime nonsuicidal self-injury	442 (84)	442 (84)	0 (0)
Recent nonsuicidal self-injury (past 4 weeks)	138 (26)	138 (26)	0 (0)
Lifetime suicidal self-injury	528 (100)	528 (100)	0 (0)
Recent suicidal self-injury (past 4 weeks)	44 (8)	44 (8)	0 (0)
Gender			
Male	84 (16)	142 (14)	191 (27)
Female	407 (77)	783 (77)	510 (72)
Other	37 (7)	88 (9)	10 (1)
Birthplace, Sweden	490 (93)	958 (95)	657 (92)
Educational level (highest)			
Elementary school	59 (11)	75 (7)	20 (3)
High school	191 (36)	355 (35)	195 (27)
University	278 (53)	583 (58)	496 (70)
Employment status before COVID-19[b]			
Student	169 (32)	358 (35)	134 (19)
Unemployed	60 (11)	112 (11)	58 (8)
Part-time employee/hourly employee	181 (34)	386 (38)	238 (33)
Full-time employee	163 (31)	337 (33)	342 (48)
Retired	75 (14)	82 (8)	75 (11)
Lifetime psychiatric disorder[b,c]			
Bipolar and related disorders	243 (46)	321 (32)	107 (15)
Major depressive disorder	517 (98)	938 (93)	515 (72)
Anxiety disorders			
Social	227 (43)	438 (43)	203 (29)
Panic	276 (52)	503 (50)	236 (33)
Generalized	253 (48)	491 (48)	248 (35)
Trauma- and stressor-related disorders	189 (36)	260 (26)	91 (13)
Feeding and eating disorders	247 (47)	383 (38)	93 (13)
Neurodevelopmental disorders	177 (34)	318 (31)	182 (26)

*Note***.** All variables are self-rated by participants at baseline. The “Other” category in gender included response options non-binary, prefer to self-define, and prefer not to answer.

[a]Individuals with a history of both nonsuicidal and suicidal self-injury are included in both groups

[b]Multiple response options allowed

[c]In total, 35 (1.9%) individuals responded “Do not know” and 106 (5.8%) responded that none of the diagnostic categories (corresponding to psychiatric disorders and neurodevelopmental disorders in the DSM) matched them
