# Supplementary material for: The trajectory of anxiety and depressive symptoms and the impact of self-injury: A longitudinal 12-month cohort study of individuals with psychiatric symptoms
Source: PLoS One. 2024 Nov 21;19(11):e0313961. doi: 10.1371/journal.pone.0313961 (PMC11581223; doi:10.1371/journal.pone.0313961)
Supplement: S2 Table — (PDF) [file pone.0313961.s003.pdf]

S2 Table

Demographic and clinical characteristics of study sample (*N* = 1810) vs. participants consenting to follow-up providing demographic and outcome data at baseline (*N* = 2308)

	Study sample (*n*=1810)	Excluded participants (*n*=2308)
**Variable**	*M (SD)*	*M (SD)*
Age†	38.07 (12.69)	34.29 (11.74)
PHQ-9 total score†	14.57 (6.61)	15.76 (6.62)
GAD-7 total score†	11.50 (5.72)	12.24 (5.70)
	n (%)	*n (%)*
Self-injury thoughts†	940 (52)	1302 (56)
Self-injury		
Lifetime nonsuicidal self-injury[a†]	1013 (56)	1369 (59)
Recent nonsuicidal self-injury (past 4 weeks)	254 (14)	374 (16)
Lifetime suicidal self-injury[b]	528 (29)	686 (30)
Recent suicidal self-injury (past 4 weeks)	44 (2)	56 (2)
Gender		
Male†	353 (20)	567 (25)
Female†	1357 (75)	1629 (71)
Other	100 (5)	112 (5)
Birthplace, Sweden	1691 (93)	2142 (93)
Educational level (highest)		
Elementary school†	98 (5)	191 (8)
High school†	577 (32)	1002 (43)
University†	1135 (63)	1115 (48)
Employment status before COVID-19[c]		
Student†	398 (22)	600 (26)
Unemployed†	179 (10)	286 (12)
Part-time employee/hourly employee	433 (24)	592 (26)
Full-time employee†	722 (40)	819 (35)
Retired†	175 (10)	156 (7)
Lifetime psychiatric disorder[c,d]		
Bipolar and related disorders	351 (19)	423 (18)
Major depressive disorder	1480 (82)	1865 (81)
Anxiety disorders		
Social	336 (19)	447 (19)
Panic†	677 (37)	1046 (45)
Generalized†	770 (43)	1104 (48)
Trauma- and stressor-related disorders	377 (21)	484 (21)
Feeding and eating disorders	407 (22)	478 (21)
Neurodevelopmental disorders†	381 (21)	583 (25)

Note. All variables are self-rated by participants at baseline. The "Other" category in gender included response options non-binary, prefer to self-define, and prefer not to answer. PHQ-9 = Patient Health Questionnaire, GAD-7 = Generalized Anxiety Disorder 7-item scale

[a]Including both past and recent nonsuicidal self-injury

[b]Including both past and recent suicidal self-injury

[c]Multiple response options allowed

[d]In total, 35 (2%) individuals responded "Do not know" and 106 (6%) responded that none of the diagnostic categories (corresponding to psychiatric disorders and neurodevelopmental disorders in the DSM) matched them among our study sample. The corresponding values for those excluded longitudinal participants providing baseline data were 36 (2%) and 136 (6%).

†Statistically significant difference in mean/proportion ($p < .05$) between Study sample and Excluded participants, as indicated by two-sample *t*-test or test of equal proportions.
