# Supplementary material for: The trajectory of anxiety and depressive symptoms and the impact of self-injury: A longitudinal 12-month cohort study of individuals with psychiatric symptoms
Source: PLoS One. 2024 Nov 21;19(11):e0313961. doi: 10.1371/journal.pone.0313961 (PMC11581223; doi:10.1371/journal.pone.0313961)
Supplement: S4 Table — (PDF) [file pone.0313961.s005.pdf]

S4 Table

Results from growth curve models with anxiety symptoms (GAD-7) as outcome

	Unadjusted growth curve				Adjusted growth curve			
**Fixed effects**	*b*	95% CI	*SE*	*p*	*b*	95% CI	*SE*	*p*
Intercept	10.71	10.47, 10.96	0.12	< .001	14.20	12.90, 15.51	0.67	< .001
Time	-0.12	-0.14, -0.10	0.01	< .001	-0.12	-0.14, -0.10	0.01	< .001
Days since study start					-0.00	-0.00, 0.00	0.00	.728
Age					-0.07	-0.08, -0.05	0.01	< .001
Gender, woman					1.11	0.53, 1.69	0.30	< .001
Gender, other					0.64	-0.45, 1.74	0.56	.251
Educational level, high school					-1.16	-2.19, -0.12	0.53	.028
Educational level, university					-2.21	-3.22, -1.21	0.51	< .001
**Random effects**	*SD*	95% CI			*SD*	95% CI		
Variance intercept	4.94	4.76, 5.12			4.76	4.58, 4.93		
Variance slope Time	0.33	0.31, 0.34			0.33	0.31, 0.34		
Residual variance	3.08	3.04, 3.11			3.08	3.04, 3.11		

Note. Reference group for gender is male and for education level elementary school. Adjusting for age, gender, educational level, and time since study start. GAD-7 = Generalized Anxiety Disorder 7-item scale.
