# Supplementary material for: The trajectory of anxiety and depressive symptoms and the impact of self-injury: A longitudinal 12-month cohort study of individuals with psychiatric symptoms
Source: PLoS One. 2024 Nov 21;19(11):e0313961. doi: 10.1371/journal.pone.0313961 (PMC11581223; doi:10.1371/journal.pone.0313961)
Supplement: S5 Table — (PDF) [file pone.0313961.s006.pdf]

S5 Table

Results from logistic growth curve models with self-injury thoughts as outcome

	Unadjusted growth curve			
**Fixed effects**	*b*	Odds ratio	*SE*	*p*
Intercept	-0.09	0.91	0.10	0.354
Time	-0.07	0.93	0.01	< .001
**Random effects**	*SD*			
Variance intercept	3.59			
Variance slope Time	0.21			
