# Supplementary material for: The trajectory of anxiety and depressive symptoms and the impact of self-injury: A longitudinal 12-month cohort study of individuals with psychiatric symptoms
Source: PLoS One. 2024 Nov 21;19(11):e0313961. doi: 10.1371/journal.pone.0313961 (PMC11581223; doi:10.1371/journal.pone.0313961)
Supplement: S6 Table — (PDF) [file pone.0313961.s007.pdf]

## S6 Table

**Proportion of individuals reporting self-injury thoughts across all time points by endorsement of self-injury (all available data in study sample)**

| | Self-injury thoughts[a] | | | | | |
|---|---|---|---|---|---|---|
| | **Suicidal self-injury**[b] ($n$ = 528) | | **Nonsuicidal self-injury**[b] ($n$ = 1013) | | **No self-injury** ($n$ = 711) | |
| **Time point** | *n* | *n* (%) | *n* | *n* (%) | *n* | *n* (%) |
| Baseline | 528 | 366 (69) | 1013 | 643 (63) | 711 | 251 (35) |
| 1 month | 478 | 318 (67) | 917 | 530 (58) | 649 | 203 (31) |
| 2 months | 463 | 293 (63) | 886 | 494 (56) | 641 | 187 (29) |
| 3 months | 467 | 312 (67) | 890 | 534 (60) | 634 | 176 (28) |
| 4 months | 464 | 303 (65) | 888 | 520 (59) | 628 | 172 (27) |
| 5 months | 474 | 313 (66) | 916 | 523 (57) | 627 | 192 (31) |
| 6 months | 446 | 300 (67) | 857 | 500 (58) | 600 | 161 (27) |
| 7 months | 440 | 282 (64) | 836 | 459 (55) | 592 | 160 (27) |
| 8 months | 443 | 286 (65) | 847 | 462 (55) | 592 | 158 (27) |
| 9 months | 442 | 293 (66) | 855 | 481 (56) | 587 | 164 (28) |
| 10 months | 423 | 266 (63) | 804 | 417 (52) | 556 | 159 (29) |
| 11 months | 408 | 249 (61) | 757 | 380 (50) | 531 | 151 (28) |
| 12 months | 373 | 247 (66) | 712 | 389 (55) | 504 | 144 (29) |

[a]Self-injury thoughts = based on PHQ-9 item 9
[b]Individuals with a history of both nonsuicidal and suicidal self-injury are included in both groups
