# Supplementary material for: The trajectory of anxiety and depressive symptoms and the impact of self-injury: A longitudinal 12-month cohort study of individuals with psychiatric symptoms
Source: PLoS One. 2024 Nov 21;19(11):e0313961. doi: 10.1371/journal.pone.0313961 (PMC11581223; doi:10.1371/journal.pone.0313961)
Supplement: S7 Table — (PDF) [file pone.0313961.s008.pdf]

S7 Table

Results from unadjusted and adjusted growth curve models with suicidal self-injury as a predictor of depression and anxiety trajectories

	Depressive symptoms				Anxiety symptoms			
Unadjusted								
**Fixed effects**	*b*	95% CI	*SE*	*p*	*b*	95% CI	*SE*	*p*
Intercept	12.95	12.63, 13.27	0.16	< .001	10.15	9.87, 10.43	0.14	< .001
Time	-0.19	-0.22, -0.17	0.01	< .001	-0.14	-0.17, -0.12	0.01	< .001
Suicidal self-injury	3.14	2.54, 3.73	0.30	< .001	1.94	1.41, 2.46	0.27	< .001
Suicidal self-injury x Time	0.08	0.03, 0.13	0.02	.001	0.07	0.03, 0.11	0.02	.001
**Random effects**	*SD*	95% CI			*SD*	95% CI		
Variance intercept	5.53	5.33, 5.73			4.86	4.68, 5.04		
Variance slope Time	0.36	0.34, 0.38			0.33	0.31, 0.34		
Residual variance	3.54	3.50, 3.57			3.08	3.04, 3.11		
Adjusted								
**Fixed effects**	*b*	95% CI	*SE*	*p*	*b*	95% CI	*SE*	*p*
Intercept	15.63	14.10, 17.16	0.78	< .001	13.10	11.78, 14.43	0.68	< .001
Time	-0.19	-0.22, -0.17	0.01	< .001	-0.14	-0.17, -0.12	0.01	< .001
Days since study start	0.00	-0.00, 0.00	0.00	.857	-0.00	-0.00, 0.00	0.00	.986
Age	-0.04	-0.06, -0.02	0.01	< .001	-0.06	-0.08, -0.05	0.01	< .001
Gender, woman	0.96	0.30, 1.62	0.34	.004	1.01	0.43, 1.58	0.29	< .001
Gender, other	0.94	-0.30, 2.19	0.64	.138	0.49	-0.59, 1.57	0.55	.375
Educational level, high school	-1.03	-2.22, 0.15	0.61	.087	-0.68	-1.70, 0.35	0.52	.197
Educational level, university	-2.42	-3.58, -1.27	0.59	< .001	-1.58	-2.58, -0.58	0.51	.002
Suicidal self-injury	2.73	2.14, 3.33	0.30	< .001	1.58	1.06, 2.10	0.26	< .001
Suicidal self-injury x Time	0.08	0.03, 0.13	0.02	.001	0.07	0.03, 0.11	0.02	.001
**Random effects**	*SD*	95% CI			*SD*	95% CI		
Variance intercept	5.42	5.22, 5.62			4.71	4.53, 4.88		
Variance slope Time	0.36	0.34, 0.38			0.33	0.31, 0.34		
Residual variance	3.54	3.50, 3.57			3.08	3.04, 3.11		

Note. Reference group for gender is male and for education level elementary school.
