# Supplementary material for: The trajectory of anxiety and depressive symptoms and the impact of self-injury: A longitudinal 12-month cohort study of individuals with psychiatric symptoms
Source: PLoS One. 2024 Nov 21;19(11):e0313961. doi: 10.1371/journal.pone.0313961 (PMC11581223; doi:10.1371/journal.pone.0313961)
Supplement: S8 Table — (PDF) [file pone.0313961.s009.pdf]

S8 Table

Results from post hoc sensitivity analyses including initial level of anxiety and depressive symptoms in the adjusted growth curve models with history of suicidal self-injury as a predictor of depression and anxiety trajectories

	Depressive symptoms				Anxiety symptoms			
Adjusted								
**Fixed effects**	*b*	95% CI	*SE*	*p*	*b*	95% CI	*SE*	*p*
Intercept	10.27	9.18, 11.36	0.56	< .001	8.82	7.86, 9.79	0.49	< .001
Time	-0.19	-0.22, -0.17	0.01	< .001	-0.14	-0.17, -0.12	0.01	< .001
Days since study start	-0.00	-0.00, -0.00	0.00	.002	-0.00	-0.00, -0.00	0.00	.002
Age	-0.02	-0.03, -0.00	0.01	.034	-0.04	-0.05, -0.02	0.01	< .001
Gender, woman	0.11	-0.36, 0.57	0.24	.655	0.30	-0.11, 0.71	0.21	.151
Gender, other	0.26	-0.61, 1.13	0.45	.561	-0.03	-0.80, 0.74	0.39	.940
Educational level, high school	-0.35	-1.17, 0.48	0.42	.416	-0.18	-0.91, 0.55	0.37	.627
Educational level, university	-1.05	-1.86, -0.24	0.41	.011	-0.56	-1.27, 0.16	0.37	.127
Clinical anxiety	2.28	1.86, 2.69	0.21	< .001	5.56	5.18, 5.94	0.19	< .001
Clinical depression	7.19	6.79, 7.60	0.20	< .001	3.00	2.65, 3.35	0.18	< .001
Suicidal self-injury	1.64	1.23, 2.04	0.21	< .001	0.75	0.39, 1.11	0.18	< .001
Suicidal self-injury x Time	0.08	0.03, 0.13	0.02	.001	0.07	0.03, 0.11	0.02	.001
**Random effects**	*SD*	95% CI			*SD*	95% CI		
Variance intercept	3.37	3.21, 3.51			3.00	2.86, 3.12		
Variance slope Time	0.36	0.34, 0.38			0.33	0.31, 0.34		
Residual variance	3.53	3.50, 3.57			3.08	3.04, 3.11		

Note. Reference group for gender is male and for education level elementary school.
