# Supplementary material for: The trajectory of anxiety and depressive symptoms and the impact of self-injury: A longitudinal 12-month cohort study of individuals with psychiatric symptoms
Source: PLoS One. 2024 Nov 21;19(11):e0313961. doi: 10.1371/journal.pone.0313961 (PMC11581223; doi:10.1371/journal.pone.0313961)
Supplement: S9 Table — (PDF) [file pone.0313961.s010.pdf]

S9 Table

Results from unadjusted and adjusted growth curve models with nonsuicidal self-injury as a predictor of depression and anxiety trajectories

	Depressive symptoms				Anxiety symptoms			
Unadjusted								
**Fixed effects**	*b*	95% CI	*SE*	*p*	*b*	95% CI	*SE*	*p*
Intercept	12.20	11.79, 12.60	0.21	< .001	9.41	9.05, 9.76	0.18	< .001
Time	-0.17	-0.21, -0.14	0.02	< .001	-0.12	-0.15, -0.09	0.01	< .001
Nonsuicidal self-injury	2.99	2.44, 3.53	0.28	< .001	2.34	1.86, 2.81	0.24	< .001
Nonsuicidal self-injury x Time	0.01	-0.04, 0.05	0.02	.718	-0.00	-0.04, 0.04	0.02	0.886
**Random effects**	*SD*	95% CI			*SD*	95% CI		
Variance intercept	5.51	5.31, 5.72			4.80	4.62, 4.98		
Variance slope Time	0.36	0.34, 0.38			0.33	0.31, 0.34		
Residual variance	3.54	3.50, 3.57			3.08	3.04, 3.11		
Adjusted								
**Fixed effects**	*b*	95% CI	*SE*	*p*	*b*	95% CI	*SE*	*p*
Intercept	14.41	12.77, 16.04	0.84	< .001	12.25	10.84, 13.66	0.72	< .001
Time	-0.17	-0.21, -0.14	0.02	< .001	-0.12	-0.15, -0.09	0.01	< .001
Days since study start	-0.00	-0.00, 0.00	0.00	.947	-0.00	-0.00, 0.00	0.00	.858
Age	-0.01	-0.03, 0.02	0.01	.582	-0.04	-0.06, -0.02	0.01	< .001
Gender, woman	0.83	0.16, 1.50	0.34	.015	0.92	0.34, 1.50	0.29	.002
Gender, other	0.37	-0.90, 1.63	0.65	.569	0.12	-0.97, 1.21	0.56	.831
Educational level, high school	-1.53	-2.72, -0.35	0.61	.011	-0.97	-2.00, 0.05	0.52	.063
Educational level, university	-3.03	-4.18, -1.88	0.59	< .001	-1.94	-2.93, -0.95	0.51	< .001
Nonsuicidal self-injury	2.61	2.01, 3.20	0.30	< .001	1.67	1.16, 2.19	0.26	< .001
Nonsuicidal self-injury x Time	0.01	-0.04, 0.05	0.02	.714	-0.00	-0.04, 0.04	0.02	.889
**Random effects**	*SD*	95% CI			*SD*	95% CI		
Variance intercept	5.44	5.24, 5.64			4.71	4.53, 4.88		
Variance slope Time	0.36	0.34, 0.38			0.33	0.31, 0.34		
Residual variance	3.54	3.50, 3.57			3.08	3.04, 3.11		

Note. Reference group for gender is male and for education level elementary school.
