# Supplementary material for: The trajectory of anxiety and depressive symptoms and the impact of self-injury: A longitudinal 12-month cohort study of individuals with psychiatric symptoms
Source: PLoS One. 2024 Nov 21;19(11):e0313961. doi: 10.1371/journal.pone.0313961 (PMC11581223; doi:10.1371/journal.pone.0313961)
Supplement: S1 Fig — (PDF) [file pone.0313961.s011.pdf]

S1 Figure

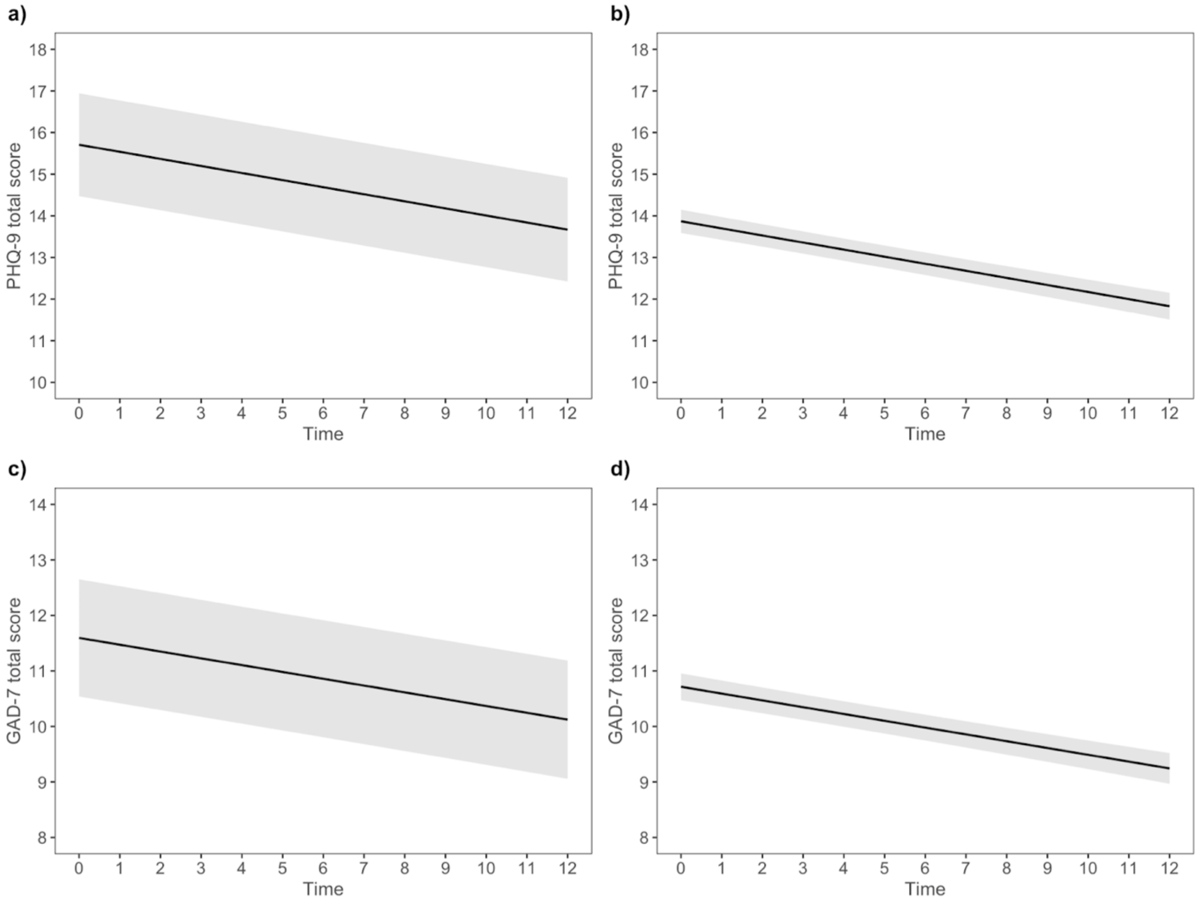

Predicted trajectories of depressive and anxiety symptoms, adjusted and unadjusted for covariates. All figures represent predicted trajectories of depression and anxiety (with 95% confidence intervals) starting at baseline (0) and the 12 months follow-up. **a)** Predicted PHQ-9 scores adjusted for covariates, **b)** Predicted PHQ-9 scores unadjusted for covariates, **c)** Predicted GAD-7 scores adjusted for covariates, **d)** Predicted GAD-7 scores unadjusted for covariates. Covariates include age, gender, educational level, and time since study start.
